# Supplementary material for: Thermodynamic and Kinetic Aspects of Gold Adsorption in Micrometric Activated Carbon and the Impact of Their Loss in Adsorption, Desorption, and Reactivation Plants
Source: Materials (Basel). 2023 Jul 12;16(14):4961. doi: 10.3390/ma16144961 (PMC10381724; doi:10.3390/ma16144961)
Supplement: Supplementary file 1 [file materials-16-04961-s001.zip › materials-2450339-supplementary.docx]

# Adsorption tests for thermodynamic and kinetic analysis

## Thermodynamic analysis of gold adsorption in CA Langmuir Isotherm

Table S1*.* Experimental data of gold adsorption in AC for the determination of the Langmuir Isotherm

| **Particle Size** | **Activate Carbon Mass** | **Ci** | **Ce** | **1/Ce** | **Log Ce** | **Ln Ce** | **qe** | **1/qe** | **Log qe** |  |
| --- | --- | --- | --- | --- | --- | --- | --- | --- | --- | --- |
| **(micron)** | **(g)** | **mg/L** | **mg/L** |  |  |  |  |  |  |  |
|  |  |  |  |  |  |  |  |  |  |  |
| 106 | 0.010 | 10 | 8.698 | 0.115 | 0.939 | 2.163 | 65.100 | 0.015 | 1.814 |  |
|  | 0.050 |  | 5.903 | 0.169 | 0.771 | 1.775 | 40.970 | 0.024 | 1.612 |  |
|  | 0.125 |  | 2.345 | 0.426 | 0.370 | 0.852 | 30.620 | 0.033 | 1.486 |  |
|  | 0.250 |  | 0.172 | 5.814 | -0.764 | -1.760 | 19.656 | 0.051 | 1.293 |  |
| 75 | 0.010 | 10 | 8.363 | 0.120 | 0.922 | 2.124 | 81.850 | 0.012 | 1.913 |  |
|  | 0.050 |  | 5.315 | 0.188 | 0.726 | 1.671 | 46.850 | 0.021 | 1.671 |  |
|  | 0.125 |  | 1.483 | 0.674 | 0.171 | 0.394 | 34.068 | 0.029 | 1.532 |  |
|  | 0.250 |  | 0.155 | 6.452 | -0.810 | -1.864 | 19.690 | 0.051 | 1.294 |  |
| 53 | 0.010 | 10 | 9.258 | 0.108 | 0.967 | 2.225 | 37.100 | 0.027 | 1.569 |  |
|  | 0.050 |  | 5.766 | 0.173 | 0.761 | 1.752 | 42.340 | 0.024 | 1.627 |  |
|  | 0.125 |  | 1.837 | 0.544 | 0.264 | 0.608 | 32.652 | 0.031 | 1.514 |  |
|  | 0.250 |  | 0.244 | 4.098 | -0.613 | -1.411 | 19.512 | 0.051 | 1.290 |  |
| 45 | 0.010 | 10 | 8.822 | 0.113 | 0.946 | 2.177 | 58.900 | 0.017 | 1.770 |  |
|  | 0.050 |  | 5.544 | 0.180 | 0.744 | 1.713 | 44.560 | 0.022 | 1.649 |  |
|  | 0.125 |  | 1.369 | 0.730 | 0.136 | 0.314 | 34.524 | 0.029 | 1.538 |  |
|  | 0.250 |  | 0.204 | 4.902 | -0.690 | -1.590 | 19.592 | 0.051 | 1.292 |  |
| 38 | 0.010 | 10 | 8.890 | 0.112 | 0.949 | 2.185 | 55.500 | 0.018 | 1.744 |  |
|  | 0.050 |  | 5.505 | 0.182 | 0.741 | 1.706 | 44.950 | 0.022 | 1.653 |  |
|  | 0.125 |  | 1.294 | 0.773 | 0.112 | 0.258 | 34.824 | 0.029 | 1.542 |  |
|  | 0.250 |  | 0.211 | 4.739 | -0.676 | -1.556 | 19.578 | 0.051 | 1.292 |  |

Table S2*.* Parameters obtained from the gold adsorption in AC for the determination of the Langmuir Isotherm (1/Ce vs 1/qe)

| **Particle Size (micron)** | **Parameters** | | | | | |
| --- | --- | --- | --- | --- | --- | --- |
|  | **b** | **m** | **qmax (mg/g)** | **KL** | **RL** | **R^2** |
| 106 | 0.0228 | 0.0049 | 43.8596 | 4.6531 | 0.0210 | 0.8175 |
| 75 | 0.0191 | 0.0050 | 52.3560 | 3.8200 | 0.0255 | 0.8706 |
| 53 | 0.0253 | 0.0064 | 39.5257 | 3.9531 | 0.0247 | 0.9737 |
| 45 | 0.0205 | 0.0063 | 48.7805 | 3.2540 | 0.0298 | 0.9499 |
| 38 | 0.0205 | 0.0065 | 48.7805 | 3.1538 | 0.0307 | 0.9679 |

Figure S1*.* Gold adsorption graphs in AC Langmuir Isotherm (1/Ce vs 1/qe)

## Thermodynamic analysis of gold adsorption in AC Temkin Isotherm

Table S3*.* Experimental data of gold adsorption in AC for the determination of the Temkin Isotherm

| **Particle Size** | **AC Mass** | **Ci** | **Ce** | **Ln Ce** | **qe** |  |
| --- | --- | --- | --- | --- | --- | --- |
| **(microns)** | **(g)** | **mg/L** | **mg/L** |  |  |  |
|  |  |  |  |  |  |  |
| 106 | 0.010 | 10 | 8.698 | 2.163 | 65.100 |  |
|  | 0.050 |  | 5.903 | 1.775 | 40.970 |  |
|  | 0.125 |  | 2.345 | 0.852 | 30.620 |  |
|  | 0.250 |  | 0.172 | -1.760 | 19.656 |  |
| 75 | 0.010 | 10 | 8.363 | 2.124 | 81.850 |  |
|  | 0.050 |  | 5.315 | 1.671 | 46.850 |  |
|  | 0.125 |  | 1.483 | 0.394 | 34.068 |  |
|  | 0.250 |  | 0.155 | -1.864 | 19.690 |  |
| 53 | 0.010 | 10 | 9.258 | 2.225 | 37.100 |  |
|  | 0.050 |  | 5.766 | 1.752 | 42.340 |  |
|  | 0.125 |  | 1.837 | 0.608 | 32.652 |  |
|  | 0.250 |  | 0.244 | -1.411 | 19.512 |  |
| 45 | 0.010 | 10 | 8.822 | 2.177 | 58.900 |  |
|  | 0.050 |  | 5.544 | 1.713 | 44.560 |  |
|  | 0.125 |  | 1.369 | 0.314 | 34.524 |  |
|  | 0.250 |  | 0.204 | -1.590 | 19.592 |  |
| 38 | 0.010 | 10 | 8.890 | 2.185 | 55.500 |  |
|  | 0.050 |  | 5.505 | 1.706 | 44.950 |  |
|  | 0.125 |  | 1.294 | 0.258 | 34.824 |  |
|  | 0.250 |  | 0.211 | -1.556 | 19.578 |  |

Table S4*.* Parameters obtained from the gold adsorption in AC for the determination of the Temkin Isotherm (Ln Ce contra qe)

| **Particle Size (microns)** | **Parameters** | | | | |
| --- | --- | --- | --- | --- | --- |
|  | **b** | **m** | **BT(J/mol)** | **KT (L/mg)** | **R^2** |
| 106 | 32.077 | 9.2522 | 9.2522 | 32.039 | 0.709 |
| 75 | 38.168 | 12.816 | 12.816 | 19.651 | 0.742 |
| 53 | 28.396 | 5.6752 | 5.6752 | 148.937 | 0.885 |
| 45 | 33.211 | 9.4598 | 9.4598 | 33.473 | 0.934 |
| 38 | 32.905 | 8.9616 | 8.9616 | 39.322 | 0.971 |

Figure S2*.* Gold adsorption graphs in AC Temkin Isotherm (Ln Ce vs qe)

## Kinetic analysis of gold adsorption in AC using the pseudo first order model

Table S5*.* Experimental data of the adsorption of gold in AC size 106 microns for the determination of the kinetic speed constant using the pseudo first order model

|  |  | **Test ID** | | | **Test ID** | | | **Test ID** | | | **Test ID** | | |
| --- | --- | --- | --- | --- | --- | --- | --- | --- | --- | --- | --- | --- | --- |
|  |  | **106-1** | | | **106-2** | | | **106-3** | | | **106-4** | | |
|  |  | **Mass** | | | **Mass** | | | **Mass** | | | **Mass** | | |
|  |  | **gr** | | | **gr** | | | **gr** | | | **gr** | | |
|  |  | 0.01 | | | 0.05 | | | 0.125 | | | 0.25 | | |
| **Time** | **Ci** | **Ce(Au)** | **qt** | **Ln(qe-qt)** | **Ce(Au)** | **qt** | **Ln(qe-qt)** | **Ce(Au)** | **qt** | **Ln(qe-qt)** | **Ce(Au)** | **qt** | **Ln(qe-qt)** |
| **min** | **mg/L** | **mg/L** | **mg/gr** |  | **mg/L** | **mg/gr** |  | **mg/L** | **mg/gr** |  | **mg/L** | **mg/gr** |  |
| 1 | 10 | 9.645 | 1.775 | 1.555 | 8.832 | 1.168 | 1.075 | 7.939 | 0.824 | 0.805 | 6.920 | 0.616 | 0.300 |
| 3 |  | 9.637 | 1.815 | 1.546 | 8.708 | 1.292 | 1.031 | 7.785 | 0.886 | 0.777 | 5.475 | 0.905 | 0.059 |
| 5 |  | 9.672 | 1.640 | 1.583 | 8.575 | 1.425 | 0.983 | 6.861 | 1.256 | 0.591 | 4.302 | 1.140 | -0.191 |
| 10 |  | 9.483 | 2.585 | 1.367 | 8.485 | 1.515 | 0.949 | 5.646 | 1.742 | 0.278 | 2.535 | 1.493 | -0.750 |
| 15 |  | 9.316 | 3.420 | 1.128 | 7.324 | 2.676 | 0.351 | 5.21 | 1.916 | 0.136 | 1.991 | 1.602 | -1.011 |
| 20 |  | 9.195 | 4.025 | 0.910 | 7.09 | 2.910 | 0.171 | 4.328 | 2.269 | -0.232 | 1.450 | 1.710 | -1.364 |
| 30 |  | 9.162 | 4.190 | 0.842 | 6.509 | 3.491 | -0.501 | 3.422 | 2.631 | -0.842 | 0.766 | 1.847 | -2.130 |
| 40 |  | 9.047 | 4.765 | 0.557 | 6.418 | 3.582 | -0.664 | 3.209 | 2.716 | -1.062 | 0.558 | 1.888 | -2.561 |
| 50 |  | 9.029 | 4.855 | 0.504 | 6.239 | 3.761 | -1.091 | 2.93 | 2.828 | -1.452 | 0.373 | 1.925 | -3.214 |
| 60 |  | 8.758 | 6.210 | -1.204 | 6.17 | 3.830 | -1.321 | 2.864 | 2.854 | -1.572 | 0.288 | 1.942 | -3.764 |
| 90 |  | 8.747 | 6.265 | -1.406 | 5.968 | 4.032 | -2.733 | 2.467 | 3.013 | -3.020 | 0.199 | 1.960 | -5.221 |
| 120 |  | 8.698 | 6.510 | - | 5.903 | 4.097 | - | 2.345 | 3.062 | - | 0.172 | 1.966 | - |
|  |  | **qe** | | | **qe** | | | **qe** | | | **qe** | | |
|  |  | **mg/gr** | | | **mg/gr** | | | **mg/gr** | | | **mg/gr** | | |
|  |  | 6.510 | | | 4.097 | | | 3.062 | | | 1.966 | | |

Table S6*.* Parameters obtained from the adsorption of gold in AC size 106 microns for the determination of the pseudo first order model (Time vs Ln (qe-qt))

| **Test ID** | **Parameters** | | | | |
| --- | --- | --- | --- | --- | --- |
|  | **b** | **m** | **qe (mg/g)** | **K1** | **R^2** |
| **106-1** | 1.7152 | -0.0354 | 5.5578 | -0.00030 | 0.9017 |
| **106-2** | 1.1155 | -0.0433 | 3.0511 | -0.00036 | 0.9846 |
| **106-3** | 0.7399 | -0.0424 | 2.0957 | -0.00035 | 0.9841 |
| **106-4** | 0.0136 | -0.0617 | 1.0137 | -0.00051 | 0.9849 |

Figure S3*.* Gold adsorption graphs in AC size 106 microns for the determination of the pseudo first order model (Time vs Ln (qe-qt))

Table S7*.* Experimental data of the adsorption of gold in AC size 75 microns for the determination of the kinetic speed constant using the pseudo first order model

|  |  | **Test ID** | | | **Test ID** | | | **Test ID** | | | **Test ID** | | |
| --- | --- | --- | --- | --- | --- | --- | --- | --- | --- | --- | --- | --- | --- |
|  |  | **75-1** | | | **75-2** | | | **75-3** | | | **75-4** | | |
|  |  | **Mass** | | | **Mass** | | | **Mass** | | | **Mass** | | |
|  |  | **gr** | | | **gr** | | | **gr** | | | **gr** | | |
|  |  | 0.01 | | | 0.05 | | | 0.125 | | | 0.25 | | |
| **Time** | **Ci** | **Ce(Au)** | **qt** | **Ln(qe-qt)** | **Ce(Au)** | **qt** | **Ln(qe-qt)** | **Ce(Au)** | **qt** | **Ln(qe-qt)** | **Ce(Au)** | **qt** | **Ln(qe-qt)** |
| **min** | **mg/L** | **mg/L** | **mg/gr** |  | **mg/L** | **mg/gr** |  | **mg/L** | **mg/gr** |  | **mg/L** | **mg/gr** |  |
| 1 | 10 | 9.234 | 3.830 | 1.471 | 7.181 | 2.819 | 0.624 | 5.972 | 1.611 | 0.585 | 2.585 | 1.483 | -0.722 |
| 3 |  | 8.783 | 6.085 | 0.742 | 7.027 | 2.973 | 0.538 | 4.678 | 2.129 | 0.245 | 2.739 | 1.452 | -0.660 |
| 5 |  | 8.582 | 7.090 | 0.091 | 6.574 | 3.426 | 0.230 | 4.523 | 2.191 | 0.196 | 3.014 | 1.397 | -0.559 |
| 10 |  | 8.531 | 7.345 | -0.174 | 6.187 | 3.813 | -0.137 | 3.103 | 2.759 | -0.434 | 1.182 | 1.764 | -1.583 |
| 15 |  | 8.529 | 7.355 | -0.186 | 6.074 | 3.926 | -0.276 | 3.369 | 2.652 | -0.282 | 0.758 | 1.848 | -2.115 |
| 20 |  | 8.501 | 7.495 | -0.371 | 5.96 | 4.040 | -0.439 | 3.005 | 2.798 | -0.496 | 0.426 | 1.915 | -2.915 |
| 30 |  | 8.464 | 7.680 | -0.683 | 5.637 | 4.363 | -1.133 | 2.292 | 3.083 | -1.128 | 0.335 | 1.933 | -3.324 |
| 40 |  | 8.461 | 7.695 | -0.713 | 5.515 | 4.485 | -1.609 | 1.995 | 3.202 | -1.586 | 0.236 | 1.953 | -4.123 |
| 50 |  | 8.414 | 7.930 | -1.366 | 5.512 | 4.488 | -1.625 | 1.623 | 3.351 | -2.882 | 0.221 | 1.956 | -4.328 |
| 60 |  | 8.413 | 7.935 | -1.386 | 5.464 | 4.536 | -1.904 | 1.621 | 3.352 | -2.897 | 0.222 | 1.956 | -4.313 |
| 90 |  | 8.39 | 8.050 | -2.002 | 5.379 | 4.621 | -2.749 | 1.524 | 3.390 | -4.110 | 0.158 | 1.968 | -7.419 |
| 120 |  | 8.363 | 8.185 | - | 5.315 | 4.685 | - | 1.483 | 3.407 | - | 0.155 | 1.969 | - |
|  |  | **qe** | | | **qe** | | | **qe** | | | **qe** | | |
|  |  | **mg/gr** | | | **mg/gr** | | | **mg/gr** | | | **mg/gr** | | |
|  |  | 8.185 | | | 4.685 | | | 3.407 | | | 1.969 | | |

Table S8*.* Parameters obtained from the adsorption of gold in CA size 75 microns for the determination of the pseudo first order model (Time vs Ln (qe-qt))

| **Test ID** | **Parameters** | | | | |
| --- | --- | --- | --- | --- | --- |
|  | **b** | **m** | **qe (mg/g)** | **K1** | **R^2** |
| **75-1** | 0.5278 | 0.0300 | 1.6952 | 0.00025 | 0.9074 |
| **75-2** | 0.2819 | -0.0687 | 1.3256 | -0.00057 | 0.9547 |
| **75-3** | 0.3588 | -0.0479 | 1.4316 | -0.00040 | 0.9239 |
| **75-4** | 0.2776 | -0.1226 | 1.3200 | -0.00102 | 0.9967 |

Figure S4*.* Gold adsorption graphs in AC size 75 microns for the determination of the pseudo first order model (Time vs Ln (qe-qt))

Table S9*.* Experimental data of the adsorption of gold in AC size 53 microns for the determination of the kinetic speed constant using the pseudo first order model

|  |  | **Test ID** | | | **Test ID** | | | **Test ID** | | | **Test ID** | | |
| --- | --- | --- | --- | --- | --- | --- | --- | --- | --- | --- | --- | --- | --- |
|  |  | **53-1** | | | **53-2** | | | **53-3** | | | **53-4** | | |
|  |  | **Mass** | | | **Mass** | | | **Mass** | | | **Mass** | | |
|  |  | **gr** | | | **gr** | | | **gr** | | | **gr** | | |
|  |  | 0.01 | | | 0.05 | | | 0.125 | | | 0.25 | | |
| **Time** | **Ci** | **Ce(Au)** | **qt** | **Ln(qe-qt)** | **Ce(Au)** | **qt** | **Ln(qe-qt)** | **Ce(Au)** | **qt** | **Ln(qe-qt)** | **Ce(Au)** | **qt** | **Ln(qe-qt)** |
| **min** | **mg/L** | **mg/L** | **mg/gr** |  | **mg/L** | **mg/gr** |  | **mg/L** | **mg/gr** |  | **mg/L** | **mg/gr** |  |
| 1 | 10 | 9.98 | 0.100 | 1.284 | 6.777 | 3.223 | 0.011 | 3.155 | 2.738 | -0.640 | 1.203 | 1.759 | -1.651 |
| 3 |  | 9.61 | 1.950 | 0.565 | 6.298 | 3.702 | -0.631 | 3.134 | 2.746 | -0.656 | 0.798 | 1.840 | -2.200 |
| 5 |  | 9.407 | 2.965 | -0.294 | 6.282 | 3.718 | -0.662 | 3.046 | 2.782 | -0.726 | 0.563 | 1.887 | -2.752 |
| 10 |  | 9.574 | 2.130 | 0.457 | 6.093 | 3.907 | -1.118 | 2.635 | 2.946 | -1.142 | 0.460 | 1.908 | -3.142 |
| 15 |  | 9.474 | 2.630 | 0.077 | 5.973 | 4.027 | -1.575 | 2.533 | 2.987 | -1.279 | 0.370 | 1.926 | -3.681 |
| 20 |  | 9.43 | 2.850 | -0.151 | 5.896 | 4.104 | -2.040 | 2.221 | 3.112 | -1.873 | 0.354 | 1.929 | -3.817 |
| 30 |  | 9.432 | 2.840 | -0.139 | 5.884 | 4.116 | -2.137 | 2.186 | 3.126 | -1.969 | 0.298 | 1.940 | -4.528 |
| 40 |  | 9.35 | 3.250 | -0.777 | 5.875 | 4.125 | -2.216 | 2.022 | 3.191 | -2.604 | 0.290 | 1.942 | -4.689 |
| 50 |  | 9.321 | 3.395 | -1.155 | 5.872 | 4.128 | -2.244 | 1.889 | 3.244 | -3.873 | 0.288 | 1.942 | -4.733 |
| 60 |  | 9.314 | 3.430 | -1.273 | 5.849 | 4.151 | -2.489 | 1.855 | 3.258 | -4.934 | 0.283 | 1.943 | -4.854 |
| 90 |  | 9.282 | 3.590 | -2.120 | 5.841 | 4.159 | -2.590 | 1.845 | 3.262 | -5.745 | 0.268 | 1.946 | -5.339 |
| 120 |  | 9.258 | 3.710 | - | 5.766 | 4.234 | - | 1.837 | 3.265 | - | 0.244 | 1.951 | - |
|  |  | **qe** | | | **qe** | | | **qe** | | | **qe** | | |
|  |  | **mg/gr** | | | **mg/gr** | | | **mg/gr** | | | **mg/gr** | | |
|  |  | 3.710 | | | 4.234 | | | 3.265 | | | 1.951 | | |

Table S10*.* Parameters obtained from the adsorption of gold in CA size 53 microns for the determination of the pseudo first order model (Time vs Ln (qe-qt))

| **Test ID** | **Parameters** | | | | |
| --- | --- | --- | --- | --- | --- |
|  | **b** | **m** | **qe (mg/g)** | **K1** | **R^2** |
| **53-1** | 0.6175 | -0.0318 | 1.8543 | -0.00027 | 0.8695 |
| **53-2** | -0.8377 | -0.0262 | 0.4327 | -0.00022 | 0.7043 |
| **53-3** | -0.4717 | -0.0625 | 0.6239 | -0.00052 | 0.9674 |
| **53-4** | -2.6544 | -0.0376 | 0.0703 | -0.00031 | 0.7790 |

Figure S5*.* Gold adsorption graphs in AC size 53 microns for the determination of the pseudo first order model (Time vs Ln (qe-qt))

Table S11*.* Experimental data of the adsorption of gold in AC size 45 microns for the determination of the kinetic speed constant using the pseudo first order model

|  |  | **Test ID** | | | **Test ID** | | | **Test ID** | | | **Test ID** | | |
| --- | --- | --- | --- | --- | --- | --- | --- | --- | --- | --- | --- | --- | --- |
|  |  | **44-1** | | | **45-2** | | | **45-3** | | | **45-4** | | |
|  |  | **Mass** | | | **Mass** | | | **Mass** | | | **Mass** | | |
|  |  | **gr** | | | **gr** | | | **gr** | | | **gr** | | |
|  |  | 0.01 | | | 0.05 | | | 0.125 | | | 0.25 | | |
| **Time** | **Ci** | **Ce(Au)** | **qt** | **Ln(qe-qt)** | **Ce(Au)** | **qt** | **Ln(qe-qt)** | **Ce(Au)** | **qt** | **Ln(qe-qt)** | **Ce(Au)** | **qt** | **Ln(qe-qt)** |
| **min** | **mg/L** | **mg/L** | **mg/gr** |  | **mg/L** | **mg/gr** |  | **mg/L** | **mg/gr** |  | **mg/L** | **mg/gr** |  |
| 1 | 10 | 9.381 | 3.095 | 1.028 | 6.318 | 3.682 | -0.256 | 3.034 | 2.786 | -0.406 | 2.063 | 1.587 | -0.989 |
| 3 |  | 8.899 | 5.505 | -0.955 | 5.796 | 4.204 | -1.378 | 2.8 | 2.880 | -0.558 | 0.813 | 1.837 | -2.105 |
| 5 |  | 8.857 | 5.715 | -1.743 | 5.741 | 4.259 | -1.625 | 2.277 | 3.089 | -1.013 | 0.523 | 1.895 | -2.752 |
| 10 |  | 8.843 | 5.785 | -2.254 | 5.817 | 4.183 | -1.298 | 1.957 | 3.217 | -1.447 | 0.318 | 1.936 | -3.781 |
| 15 |  | 8.8 | 6.000 | - | 5.681 | 4.319 | -1.988 | 1.762 | 3.295 | -1.850 | 0.260 | 1.948 | -4.492 |
| 20 |  | 8.795 | 6.025 | - | 5.65 | 4.350 | -2.244 | 1.637 | 3.345 | -2.233 | 0.248 | 1.950 | -4.733 |
| 30 |  | 8.749 | 6.255 | - | 5.575 | 4.425 | -3.474 | 1.526 | 3.390 | -2.768 | 0.217 | 1.957 | -5.952 |
| 40 |  | 8.9 | 5.500 | -0.942 | 5.771 | 4.229 | -1.483 | 1.478 | 3.409 | -3.133 | 0.207 | 1.959 | -7.419 |
| 50 |  | 8.895 | 5.525 | -1.008 | 5.611 | 4.389 | -2.703 | 1.518 | 3.393 | -2.820 | 0.196 | 1.961 | - |
| 60 |  | 8.817 | 5.915 | - | 5.594 | 4.406 | -2.996 | 1.373 | 3.451 | -6.438 | 0.192 | 1.962 | - |
| 90 |  | 8.857 | 5.715 | -1.743 | 5.613 | 4.387 | -2.674 | 1.372 | 3.451 | -6.725 | 0.212 | 1.958 | -6.438 |
| 120 |  | 8.822 | 5.890 | - | 5.544 | 4.456 | - | 1.369 | 3.452 | - | 0.204 | 1.959 | - |
|  |  | **qe** | | | **qe** | | | **qe** | | | **qe** | | |
|  |  | **mg/gr** | | | **mg/gr** | | | **mg/gr** | | | **mg/gr** | | |
|  |  | 5.890 | | | 4.456 | | | 3.452 | | | 1.959 | | |

Table S12*.* Parameters obtained from the adsorption of gold in CA size 45 microns for the determination of the pseudo first order model (Time vs Ln (qe-qt))

| **Test ID** | **Parameters** | | | | |
| --- | --- | --- | --- | --- | --- |
|  | **b** | **m** | **qe (mg/g)** | **K1** | **R^2** |
| **44-1** | 0.5532 | -0.323 | 1.7388 | -0.0027 | 0.7484 |
| **45-2** | -1.3866 | -0.0212 | 0.2499 | -0.0002 | 0.4183 |
| **45-3** | -0.5542 | -0.0719 | 0.5745 | -0.0006 | 0.898 |
| **45-4** | -1.7486 | -0.1471 | 0.1740 | -0.0012 | 0.9478 |

Figure S6*.* Gold adsorption graphs in AC size 45 microns for the determination of the pseudo first order model (Time vs Ln (qe-qt))

Table S13*.* Experimental data of the adsorption of gold in AC size 38 microns for the determination of the kinetic speed constant using the pseudo first order model

|  |  | **Test ID** | | | **Test ID** | | | **Test ID** | | | **Test ID** | | |
| --- | --- | --- | --- | --- | --- | --- | --- | --- | --- | --- | --- | --- | --- |
|  |  | **38-1** | | | **38-2** | | | **38-3** | | | **38-4** | | |
|  |  | **Mass** | | | **Mass** | | | **Mass** | | | **Mass** | | |
|  |  | **gr** | | | **gr** | | | **gr** | | | **gr** | | |
|  |  | 0.01 | | | 0.05 | | | 0.125 | | | 0.25 | | |
| **Time** | **Ci** | **Ce(Au)** | **qt** | **Ln(qe-qt)** | **Ce(Au)** | **qt** | **Ln(qe-qt)** | **Ce(Au)** | **qt** | **Ln(qe-qt)** | **Ce(Au)** | **qt** | **Ln(qe-qt)** |
| **min** | **mg/L** | **mg/L** | **mg/gr** |  | **mg/L** | **mg/gr** |  | **mg/L** | **mg/gr** |  | **mg/L** | **mg/gr** |  |
| 1 | 10 | 9.086 | 4.570 | -0.020 | 5.939 | 4.061 | -0.835 | 2.149 | 3.140 | -1.073 | 1.580 | 1.684 | -1.295 |
| 3 |  | 8.872 | 5.640 | - | 5.66 | 4.340 | -1.864 | 1.8 | 3.280 | -1.598 | 0.749 | 1.850 | -2.229 |
| 5 |  | 8.862 | 5.690 | - | 5.619 | 4.381 | -2.172 | 1.732 | 3.307 | -1.742 | 0.494 | 1.901 | -2.872 |
| 10 |  | 8.933 | 5.335 | -1.537 | 5.596 | 4.404 | -2.397 | 1.714 | 3.314 | -1.784 | 0.373 | 1.925 | -3.430 |
| 15 |  | 9.003 | 4.985 | -0.571 | 5.47 | 4.530 | - | 1.46 | 3.416 | -2.712 | 0.290 | 1.942 | -4.148 |
| 20 |  | 8.995 | 5.025 | -0.644 | 5.437 | 4.563 | - | 1.427 | 3.429 | -2.934 | 0.267 | 1.947 | -4.492 |
| 30 |  | 8.862 | 5.690 | - | 5.634 | 4.366 | -2.048 | 1.493 | 3.403 | -2.531 | 0.258 | 1.948 | -4.667 |
| 40 |  | 8.86 | 5.700 | - | 5.429 | 4.571 | - | 1.472 | 3.411 | -2.642 | 0.241 | 1.952 | -5.116 |
| 50 |  | 8.85 | 5.750 | - | 5.469 | 4.531 | - | 1.355 | 3.458 | -3.713 | 0.245 | 1.951 | -4.991 |
| 60 |  | 8.912 | 5.440 | -2.207 | 5.502 | 4.498 | - | 1.369 | 3.452 | -3.507 | 0.228 | 1.954 | -5.684 |
| 90 |  | 8.911 | 5.445 | -2.254 | 5.446 | 4.554 | - | 1.584 | 3.366 | -2.154 | 0.226 | 1.955 | -5.809 |
| 120 |  | 8.89 | 5.550 | - | 5.505 | 4.495 | - | 1.294 | 3.482 | - | 0.211 | 1.958 | - |
|  |  | **qe** | | | **qe** | | | **qe** | | | **qe** | | |
|  |  | **mg/gr** | | | **mg/gr** | | | **mg/gr** | | | **mg/gr** | | |
|  |  | 5.550 | | | 4.495 | | | 3.482 | | | 1.958 | | |

Table S14*.* Parameters obtained from the adsorption of gold in CA size 38 microns for the determination of the pseudo first order model (Time vs Ln (qe-qt))

| **Test ID** | **Parameters** | | | | |
| --- | --- | --- | --- | --- | --- |
|  | **b** | **m** | **qe (mg/g)** | **K1** | **R^2** |
| **38-1** | -0.0658 | -0.0201 | 0.9363 | -0.00017 | 0.3851 |
| **38-2** | -1.1021 | -0.1505 | 0.3322 | -0.00125 | 0.709 |
| **38-3** | -1.93 | -0.0159 | 0.1451 | -0.00013 | 0.3002 |
| **38-4** | -2.7675 | -0.0441 | 0.0628 | -0.00037 | 0.7318 |

Figure S7*.* Gold adsorption graphs in AC size 38 microns for the determination of the pseudo first order model (Time vs Ln (qe-qt))

## Kinetic analysis of gold adsorption in AC using the pseudo second order model

Table S15*.* Experimental data of the adsorption of gold in AC size 106 microns for the determination of the kinetic speed constant using the pseudo second order model

|  |  | **Test ID** | | | **Test ID** | | | **Test ID** | | | **Test ID** | | |
| --- | --- | --- | --- | --- | --- | --- | --- | --- | --- | --- | --- | --- | --- |
|  |  | **106-1** | | | **106-2** | | | **106-3** | | | **106-4** | | |
|  |  | **Mass** | | | **Mass** | | | **Mass** | | | **Mass** | | |
|  |  | **gr** | | | **gr** | | | **gr** | | | **gr** | | |
|  |  | 0.01 | | | 0.05 | | | 0.125 | | | 0.25 | | |
| **Time** | **Ci** | **Ce(Au)** | **qt** | **t/qt** | **Ce(Au)** | **qt** | **t/qt** | **Ce(Au)** | **qt** | **t/qt** | **Ce(Au)** | **qt** | **t/qt** |
| **min** | **mg/L** | **mg/L** | **mg/gr** |  | **mg/L** | **mg/gr** |  | **mg/L** | **mg/gr** |  | **mg/L** | **mg/gr** |  |
| 1 | 10 | 9.645 | 1.775 | 0.563 | 8.832 | 1.168 | 0.856 | 7.939 | 0.824 | 1.213 | 6.920 | 0.616 | 1.623 |
| 3 |  | 9.637 | 1.815 | 1.653 | 8.708 | 1.292 | 2.322 | 7.785 | 0.886 | 3.386 | 5.475 | 0.905 | 3.315 |
| 5 |  | 9.672 | 1.640 | 3.049 | 8.575 | 1.425 | 3.509 | 6.861 | 1.256 | 3.982 | 4.302 | 1.140 | 4.388 |
| 10 |  | 9.483 | 2.585 | 3.868 | 8.485 | 1.515 | 6.601 | 5.646 | 1.742 | 5.742 | 2.535 | 1.493 | 6.698 |
| 15 |  | 9.316 | 3.420 | 4.386 | 7.324 | 2.676 | 5.605 | 5.21 | 1.916 | 7.829 | 1.991 | 1.602 | 9.364 |
| 20 |  | 9.195 | 4.025 | 4.969 | 7.09 | 2.910 | 6.873 | 4.328 | 2.269 | 8.815 | 1.450 | 1.710 | 11.696 |
| 30 |  | 9.162 | 4.190 | 7.160 | 6.509 | 3.491 | 8.594 | 3.422 | 2.631 | 11.402 | 0.766 | 1.847 | 16.244 |
| 40 |  | 9.047 | 4.765 | 8.395 | 6.418 | 3.582 | 11.167 | 3.209 | 2.716 | 14.725 | 0.558 | 1.888 | 21.182 |
| 50 |  | 9.029 | 4.855 | 10.299 | 6.239 | 3.761 | 13.294 | 2.93 | 2.828 | 17.680 | 0.373 | 1.925 | 25.969 |
| 60 |  | 8.758 | 6.210 | 9.662 | 6.17 | 3.830 | 15.666 | 2.864 | 2.854 | 21.020 | 0.288 | 1.942 | 30.890 |
| 90 |  | 8.747 | 6.265 | 14.366 | 5.968 | 4.032 | 22.321 | 2.467 | 3.013 | 29.869 | 0.199 | 1.960 | 45.914 |
| 120 |  | 8.698 | 6.510 | 18.433 | 5.903 | 4.097 | 29.290 | 2.345 | 3.062 | 39.190 | 0.172 | 1.966 | 61.050 |
|  |  | **qe** | | | **qe** | | | **qe** | | | **qe** | | |
|  |  | **mg/gr** | | | **mg/gr** | | | **mg/gr** | | | **mg/gr** | | |
|  |  | 6.510 | | | 4.097 | | | 3.062 | | | 1.966 | | |

Table S16*.* Parameters obtained from the adsorption of gold in CA size 106 microns for the determination of the pseudo second order model (Time vs t/qt)

| **Test ID** | **Parameters** | | | | | |
| --- | --- | --- | --- | --- | --- | --- |
|  | **b** | **m** | **qe (mg/g)** | **qe^2 (mg/g)** | **K2** | **R^2** |
| **106-1** | 2.0474 | 0.1402 | 7.1327 | 50.8749 | 0.0096 | 0.9760 |
| **106-2** | 2.1641 | 0.2255 | 4.4346 | 19.6656 | 0.0235 | 0.9904 |
| **106-3** | 2.3488 | 0.3078 | 3.2489 | 10.5551 | 0.0403 | 0.9978 |
| **106-4** | 1.6362 | 0.4926 | 2.0300 | 4.1211 | 0.1483 | 0.9998 |

Figure S8*.* Graphs of gold adsorption in CA size 106 microns for the determination of the pseudo second order model (Time vs t/qt)

Table S17*.* Experimental data of the adsorption of gold in AC size 75 microns for the determination of the kinetic speed constant using the pseudo second order model

|  |  | **Test ID** | | | **Test ID** | | | **Test ID** | | | **Test ID** | | |
| --- | --- | --- | --- | --- | --- | --- | --- | --- | --- | --- | --- | --- | --- |
|  |  | **75-1** | | | **75-2** | | | **75-3** | | | **75-4** | | |
|  |  | **Mass** | | | **Mass** | | | **Mass** | | | **Mass** | | |
|  |  | **gr** | | | **gr** | | | **gr** | | | **gr** | | |
|  |  | 0.01 | | | 0.05 | | | 0.125 | | | 0.25 | | |
| **Time** | **Ci** | **Ce(Au)** | **qt** | **t/qt** | **Ce(Au)** | **qt** | **t/qt** | **Ce(Au)** | **qt** | **t/qt** | **Ce(Au)** | **qt** | **t/qt** |
| **min** | **mg/L** | **mg/L** | **mg/gr** |  | **mg/L** | **mg/gr** |  | **mg/L** | **mg/gr** |  | **mg/L** | **mg/gr** |  |
| 1 | 10 | 9.234 | 3.830 | 0.261 | 7.181 | 2.819 | 0.355 | 5.972 | 1.611 | 0.621 | 2.585 | 1.483 | 0.674 |
| 3 |  | 8.783 | 6.085 | 0.493 | 7.027 | 2.973 | 1.009 | 4.678 | 2.129 | 1.409 | 2.739 | 1.452 | 2.066 |
| 5 |  | 8.582 | 7.090 | 0.705 | 6.574 | 3.426 | 1.459 | 4.523 | 2.191 | 2.282 | 3.014 | 1.397 | 3.579 |
| 10 |  | 8.531 | 7.345 | 1.361 | 6.187 | 3.813 | 2.623 | 3.103 | 2.759 | 3.625 | 1.182 | 1.764 | 5.670 |
| 15 |  | 8.529 | 7.355 | 2.039 | 6.074 | 3.926 | 3.821 | 3.369 | 2.652 | 5.655 | 0.758 | 1.848 | 8.115 |
| 20 |  | 8.501 | 7.495 | 2.668 | 5.96 | 4.040 | 4.950 | 3.005 | 2.798 | 7.148 | 0.426 | 1.915 | 10.445 |
| 30 |  | 8.464 | 7.680 | 3.906 | 5.637 | 4.363 | 6.876 | 2.292 | 3.083 | 9.730 | 0.335 | 1.933 | 15.520 |
| 40 |  | 8.461 | 7.695 | 5.198 | 5.515 | 4.485 | 8.919 | 1.995 | 3.202 | 12.492 | 0.236 | 1.953 | 20.483 |
| 50 |  | 8.414 | 7.930 | 6.305 | 5.512 | 4.488 | 11.141 | 1.623 | 3.351 | 14.922 | 0.221 | 1.956 | 25.565 |
| 60 |  | 8.413 | 7.935 | 7.561 | 5.464 | 4.536 | 13.228 | 1.621 | 3.352 | 17.902 | 0.222 | 1.956 | 30.681 |
| 90 |  | 8.39 | 8.050 | 11.180 | 5.379 | 4.621 | 19.476 | 1.524 | 3.390 | 26.546 | 0.158 | 1.968 | 45.722 |
| 120 |  | 8.363 | 8.185 | 14.661 | 5.315 | 4.685 | 25.614 | 1.483 | 3.407 | 35.224 | 0.155 | 1.969 | 60.945 |
|  |  | **qe** | | | **qe** | | | **qe** | | | **qe** | | |
|  |  | **mg/gr** | | | **mg/gr** | | | **mg/gr** | | | **mg/gr** | | |
|  |  | 8.185 | | | 4.685 | | | 3.407 | | | 1.969 | | |

Table S18*.* Parameters obtained from the adsorption of gold in CA size 75 microns for the determination of the pseudo second order model (Time vs t/qt)

| **Test ID** | **Parameters** | | | | | |
| --- | --- | --- | --- | --- | --- | --- |
|  | **b** | **m** | **qe (mg/g)** | **qe^2 (mg/g)** | **K2** | **R^2** |
| **75-1** | 0.1928 | 0.1217 | 8.2169 | 67.5179 | 0.0768 | 0.9997 |
| **75-2** | 0.4896 | 0.2108 | 4.7438 | 22.5040 | 0.0908 | 0.9996 |
| **75-3** | 0.8691 | 0.2863 | 3.4928 | 12.1999 | 0.0943 | 0.9991 |
| **75-4** | 0.5303 | 0.5025 | 1.9900 | 3.9603 | 0.4762 | 0.9999 |

Figure S9*.* Graphs of gold adsorption in CA size 75 microns for the determination of the pseudo second order model (Time vs t/qt)

Table S19*.* Experimental data of the adsorption of gold in AC size 53 microns for the determination of the kinetic speed constant using the pseudo second order model

|  |  | **Test ID** | | | **Test ID** | | | **Test ID** | | | **Test ID** | | |
| --- | --- | --- | --- | --- | --- | --- | --- | --- | --- | --- | --- | --- | --- |
|  |  | **53-1** | | | **53-2** | | | **53-3** | | | **53-4** | | |
|  |  | **Mass** | | | **Mass** | | | **Mass** | | | **Mass** | | |
|  |  | **gr** | | | **gr** | | | **gr** | | | **gr** | | |
|  |  | 0.01 | | | 0.05 | | | 0.125 | | | 0.25 | | |
| **Time** | **Ci** | **Ce(Au)** | **qt** | **t/qt** | **Ce(Au)** | **qt** | **t/qt** | **Ce(Au)** | **qt** | **t/qt** | **Ce(Au)** | **qt** | **t/qt** |
| **min** | **mg/L** | **mg/L** | **mg/gr** |  | **mg/L** | **mg/gr** |  | **mg/L** | **mg/gr** |  | **mg/L** | **mg/gr** |  |
| 1 | 10 | 9.98 | 0.100 | 10.000 | 6.777 | 3.223 | 0.310 | 3.155 | 2.738 | 0.365 | 1.203 | 1.759 | 0.568 |
| 3 |  | 9.61 | 1.950 | 1.538 | 6.298 | 3.702 | 0.810 | 3.134 | 2.746 | 1.092 | 0.798 | 1.840 | 1.630 |
| 5 |  | 9.407 | 2.965 | 1.686 | 6.282 | 3.718 | 1.345 | 3.046 | 2.782 | 1.798 | 0.563 | 1.887 | 2.649 |
| 10 |  | 9.574 | 2.130 | 4.695 | 6.093 | 3.907 | 2.560 | 2.635 | 2.946 | 3.394 | 0.460 | 1.908 | 5.241 |
| 15 |  | 9.474 | 2.630 | 5.703 | 5.973 | 4.027 | 3.725 | 2.533 | 2.987 | 5.022 | 0.370 | 1.926 | 7.788 |
| 20 |  | 9.43 | 2.850 | 7.018 | 5.896 | 4.104 | 4.873 | 2.221 | 3.112 | 6.428 | 0.354 | 1.929 | 10.367 |
| 30 |  | 9.432 | 2.840 | 10.563 | 5.884 | 4.116 | 7.289 | 2.186 | 3.126 | 9.598 | 0.298 | 1.940 | 15.461 |
| 40 |  | 9.35 | 3.250 | 12.308 | 5.875 | 4.125 | 9.697 | 2.022 | 3.191 | 12.534 | 0.290 | 1.942 | 20.597 |
| 50 |  | 9.321 | 3.395 | 14.728 | 5.872 | 4.128 | 12.112 | 1.889 | 3.244 | 15.411 | 0.288 | 1.942 | 25.741 |
| 60 |  | 9.314 | 3.430 | 17.493 | 5.849 | 4.151 | 14.454 | 1.855 | 3.258 | 18.416 | 0.283 | 1.943 | 30.874 |
| 90 |  | 9.282 | 3.590 | 25.070 | 5.841 | 4.159 | 21.640 | 1.845 | 3.262 | 27.590 | 0.268 | 1.946 | 46.239 |
| 120 |  | 9.258 | 3.710 | 32.345 | 5.766 | 4.234 | 28.342 | 1.837 | 3.265 | 36.751 | 0.244 | 1.951 | 61.501 |
|  |  | **qe** | | | **qe** | | | **qe** | | | **qe** | | |
|  |  | **mg/gr** | | | **mg/gr** | | | **mg/gr** | | | **mg/gr** | | |
|  |  | 3.710 | | | 4.234 | | | 3.265 | | | 1.951 | | |

Table S20*.* Parameters obtained from the adsorption of gold in CA size 53 microns for the determination of the pseudo second order model (Time vs t/qt)

| **Test ID** | **Parameters** | | | | | |
| --- | --- | --- | --- | --- | --- | --- |
|  | **b** | **m** | **qe (mg/g)** | **qe^2 (mg/g)** | **K2** | **R^2** |
| **53-1** | 1.5994 | 0.2608 | 3.8344 | 14.7023 | 0.0425 | 0.9956 |
| **53-2** | 0.1746 | 0.2366 | 4.2265 | 17.8637 | 0.3206 | 0.9999 |
| **53-3** | 0.2939 | 0.3038 | 3.2916 | 10.8349 | 0.3140 | 0.9999 |
| **53-4** | 0.1045 | 0.5122 | 1.9524 | 3.8117 | 2.5105 | 1.0000 |

Figure S10*.* Graphs of gold adsorption in CA size 53 microns for the determination of the pseudo second order model (Time vs t/qt)

Table S21*.* Experimental data of the adsorption of gold in AC size 45 microns for the determination of the kinetic speed constant using the pseudo second order model

|  |  | **Test ID** | | | **Test ID** | | | **Test ID** | | | **Test ID** | | |
| --- | --- | --- | --- | --- | --- | --- | --- | --- | --- | --- | --- | --- | --- |
|  |  | **44-1** | | | **45-2** | | | **45-3** | | | **45-4** | | |
|  |  | **Mass** | | | **Mass** | | | **Mass** | | | **Mass** | | |
|  |  | **gr** | | | **gr** | | | **gr** | | | **gr** | | |
|  |  | 0.01 | | | 0.05 | | | 0.125 | | | 0.25 | | |
| **Time** | **Ci** | **Ce(Au)** | **qt** | **t/qt** | **Ce(Au)** | **qt** | **t/qt** | **Ce(Au)** | **qt** | **t/qt** | **Ce(Au)** | **qt** | **t/qt** |
| **min** | **mg/L** | **mg/L** | **mg/gr** |  | **mg/L** | **mg/gr** |  | **mg/L** | **mg/gr** |  | **mg/L** | **mg/gr** |  |
| 1 | 10 | 9.381 | 3.095 | 0.323 | 6.318 | 3.682 | 0.272 | 3.034 | 2.786 | 0.359 | 2.063 | 1.587 | 0.630 |
| 3 |  | 8.899 | 5.505 | 0.545 | 5.796 | 4.204 | 0.714 | 2.8 | 2.880 | 1.042 | 0.813 | 1.837 | 1.633 |
| 5 |  | 8.857 | 5.715 | 0.875 | 5.741 | 4.259 | 1.174 | 2.277 | 3.089 | 1.619 | 0.523 | 1.895 | 2.638 |
| 10 |  | 8.843 | 5.785 | 1.729 | 5.817 | 4.183 | 2.391 | 1.957 | 3.217 | 3.108 | 0.318 | 1.936 | 5.164 |
| 15 |  | 8.8 | 6.000 | 2.500 | 5.681 | 4.319 | 3.473 | 1.762 | 3.295 | 4.552 | 0.260 | 1.948 | 7.700 |
| 20 |  | 8.795 | 6.025 | 3.320 | 5.65 | 4.350 | 4.598 | 1.637 | 3.345 | 5.979 | 0.248 | 1.950 | 10.254 |
| 30 |  | 8.749 | 6.255 | 4.796 | 5.575 | 4.425 | 6.780 | 1.526 | 3.390 | 8.851 | 0.217 | 1.957 | 15.333 |
| 40 |  | 8.9 | 5.500 | 7.273 | 5.771 | 4.229 | 9.459 | 1.478 | 3.409 | 11.734 | 0.207 | 1.959 | 20.423 |
| 50 |  | 8.895 | 5.525 | 9.050 | 5.611 | 4.389 | 11.392 | 1.518 | 3.393 | 14.737 | 0.196 | 1.961 | 25.500 |
| 60 |  | 8.817 | 5.915 | 10.144 | 5.594 | 4.406 | 13.618 | 1.373 | 3.451 | 17.387 | 0.192 | 1.962 | 30.587 |
| 90 |  | 8.857 | 5.715 | 15.748 | 5.613 | 4.387 | 20.515 | 1.372 | 3.451 | 26.078 | 0.212 | 1.958 | 45.975 |
| 120 |  | 8.822 | 5.890 | 20.374 | 5.544 | 4.456 | 26.930 | 1.369 | 3.452 | 34.758 | 0.204 | 1.959 | 61.249 |
|  |  | **qe** | | | **qe** | | | **qe** | | | **qe** | | |
|  |  | **mg/gr** | | | **mg/gr** | | | **mg/gr** | | | **mg/gr** | | |
|  |  | 5.890 | | | 4.456 | | | 3.452 | | | 1.959 | | |

Table S22*.* Parameters obtained from the adsorption of gold in CA size 45 microns for the determination of the pseudo second order model (Time vs t/qt)

| **Test ID** | **Parameters** | | | | | |
| --- | --- | --- | --- | --- | --- | --- |
|  | **b** | **m** | **qe (mg/g)** | **qe^2 (mg/g)** | **K2** | **R^2** |
| **44-1** | 0.0164 | 0.1719 | 5.8173 | 33.8414 | 1.8018 | 0.9984 |
| **45-2** | 0.1135 | 0.2251 | 4.4425 | 19.7355 | 0.4464 | 0.9997 |
| **45-3** | 0.1906 | 0.2881 | 3.4710 | 12.0480 | 0.4355 | 1.0000 |
| **45-4** | 0.0701 | 0.5096 | 1.9623 | 3.8507 | 3.7046 | 1.0000 |

Figure S11*.* Graphs of gold adsorption in CA size 106 microns for the determination of the pseudo second order model (Time vs t/qt)

Table S23*.* Experimental data of the adsorption of gold in AC size 38 microns for the determination of the kinetic speed constant using the pseudo second order model

|  |  | **Test ID** | | | **Test ID** | | | **Test ID** | | | **Test ID** | | |
| --- | --- | --- | --- | --- | --- | --- | --- | --- | --- | --- | --- | --- | --- |
|  |  | **38-1** | | | **38-2** | | | **38-3** | | | **38-4** | | |
|  |  | **Mass** | | | **Mass** | | | **Mass** | | | **Mass** | | |
|  |  | **gr** | | | **gr** | | | **gr** | | | **gr** | | |
|  |  | 0.01 | | | 0.05 | | | 0.125 | | | 0.25 | | |
| **Time** | **Ci** | **Ce(Au)** | **qt** | **t/qt** | **Ce(Au)** | **qt** | **t/qt** | **Ce(Au)** | **qt** | **t/qt** | **Ce(Au)** | **qt** | **t/qt** |
| **min** | **mg/L** | **mg/L** | **mg/gr** |  | **mg/L** | **mg/gr** |  | **mg/L** | **mg/gr** |  | **mg/L** | **mg/gr** |  |
| 1 | 10 | 9.086 | 4.570 | 0.219 | 5.939 | 4.061 | 0.246 | 2.149 | 3.140 | 0.318 | 1.580 | 1.684 | 0.594 |
| 3 |  | 8.872 | 5.640 | 0.532 | 5.66 | 4.340 | 0.691 | 1.8 | 3.280 | 0.915 | 0.749 | 1.850 | 1.621 |
| 5 |  | 8.862 | 5.690 | 0.879 | 5.619 | 4.381 | 1.141 | 1.732 | 3.307 | 1.512 | 0.494 | 1.901 | 2.630 |
| 10 |  | 8.933 | 5.335 | 1.874 | 5.596 | 4.404 | 2.271 | 1.714 | 3.314 | 3.017 | 0.373 | 1.925 | 5.194 |
| 15 |  | 9.003 | 4.985 | 3.009 | 5.47 | 4.530 | 3.311 | 1.46 | 3.416 | 4.391 | 0.290 | 1.942 | 7.724 |
| 20 |  | 8.995 | 5.025 | 3.980 | 5.437 | 4.563 | 4.383 | 1.427 | 3.429 | 5.832 | 0.267 | 1.947 | 10.274 |
| 30 |  | 8.862 | 5.690 | 5.272 | 5.634 | 4.366 | 6.871 | 1.493 | 3.403 | 8.816 | 0.258 | 1.948 | 15.397 |
| 40 |  | 8.86 | 5.700 | 7.018 | 5.429 | 4.571 | 8.751 | 1.472 | 3.411 | 11.726 | 0.241 | 1.952 | 20.494 |
| 50 |  | 8.85 | 5.750 | 8.696 | 5.469 | 4.531 | 11.035 | 1.355 | 3.458 | 14.459 | 0.245 | 1.951 | 25.628 |
| 60 |  | 8.912 | 5.440 | 11.029 | 5.502 | 4.498 | 13.339 | 1.369 | 3.452 | 17.379 | 0.228 | 1.954 | 30.700 |
| 90 |  | 8.911 | 5.445 | 16.529 | 5.446 | 4.554 | 19.763 | 1.584 | 3.366 | 26.735 | 0.226 | 1.955 | 46.041 |
| 120 |  | 8.89 | 5.550 | 21.622 | 5.505 | 4.495 | 26.696 | 1.294 | 3.482 | 34.459 | 0.211 | 1.958 | 61.293 |
|  |  | **qe** | | | **qe** | | | **qe** | | | **qe** | | |
|  |  | **mg/gr** | | | **mg/gr** | | | **mg/gr** | | | **mg/gr** | | |
|  |  | 5.550 | | | 4.495 | | | 3.482 | | | 1.958 | | |

Table S24*.* Parameters obtained from the adsorption of gold in CA size 38 microns for the determination of the pseudo second order model (Time vs t/qt)

| **Test ID** | **Parameters** | | | | | |
| --- | --- | --- | --- | --- | --- | --- |
|  | **b** | **m** | **qe (mg/g)** | **qe^2 (mg/g)** | **K2** | **R^2** |
| **38-1** | 0.0491 | 0.1803 | 5.5463 | 30.7616 | 0.6621 | 0.9990 |
| **38-2** | 0.0226 | 0.2212 | 4.5208 | 20.4376 | 2.1650 | 0.9998 |
| **38-3** | 0.0792 | 0.2897 | 3.4518 | 11.9152 | 1.0597 | 0.9996 |
| **38-4** | 0.0847 | 0.5103 | 1.9596 | 3.8402 | 3.0745 | 1.0000 |

Figure S12*.* Graphs of gold adsorption in CA size 106 microns for the determination of the pseudo second order model (Time vs t/qt)
